# Temporal and spatial transcriptomic dynamics across brain development in *Xenopus laevis* tadpoles

**DOI:** 10.1093/g3journal/jkab387

**Published:** 2021-11-09

**Authors:** Aaron C Ta, Lin-Chien Huang, Caroline R McKeown, Jennifer E Bestman, Kendall Van Keuren-Jensen, Hollis T Cline

**Affiliations:** 1 Neuroscience Department and The Dorris Neuroscience Center, The Scripps Research Institute, La Jolla, CA 92037, USA; 2 Department of Neuroscience, University of California, San Diego, La Jolla, CA 92037, USA; 3 Neurogenomics Division, Translational Genomics Research Institute, Phoenix, AZ 85004, USA

**Keywords:** brain development, neuron, neural progenitor cell, *Xenopus*, transcriptome, differential expression, premetamorphosis

## Abstract

Amphibian metamorphosis is a transitional period that involves significant changes in the cell-type populations and biological processes occurring in the brain. Analysis of gene expression dynamics during this process may provide insight into the molecular events underlying these changes. We conducted differential gene expression analyses of the developing *Xenopus laevis* tadpole brain during this period in two ways: first, over stages of the development in the midbrain and, second, across regions of the brain at a single developmental stage. We found that genes pertaining to positive regulation of neural progenitor cell proliferation as well as known progenitor cell markers were upregulated in the midbrain prior to metamorphic climax; concurrently, expression of cell cycle timing regulators decreased across this period, supporting the notion that cell cycle lengthening contributes to a decrease in proliferation by the end of metamorphosis. We also found that at the start of metamorphosis, neural progenitor populations appeared to be similar across the fore-, mid-, and hindbrain regions. Genes pertaining to negative regulation of differentiation were upregulated in the spinal cord compared to the rest of the brain, however, suggesting that different programs may regulate neurogenesis there. Finally, we found that regulation of biological processes like cell fate commitment and synaptic signaling follow similar trajectories in the brain across early tadpole metamorphosis and mid- to late-embryonic mouse development. By comparing expression across both temporal and spatial conditions, we have been able to illuminate cell-type and biological pathway dynamics in the brain during metamorphosis.

## Teaser

Amphibian metamorphosis involves significant changes in cell type populations and biological processes in the brain. To identify these changes, gene expression analyses of the *Xenopus laevis* brain was performed temporally over stages of midbrain development and spatially across brain regions from a single stage. Expression data was also compared between tadpole and mouse, identifying similarities in brain developmental stages. Comparing expression across both temporal and spatial conditions helped elucidate cell type and biological pathway dynamics in the brain during metamorphosis.

## Introduction

*Xenopus laevis*, the African clawed frog is a resource-efficient model organism popular for neurobiological research (Pratt and Khakhalin 2013; Blum and Ott 2018). Gene expression in the entire *X. laevis* body at successive stages of early development has been well-documented in the literature (Pratt and Khakhalin 2013; Owens *et al.* 2016; Session *et al.* 2016), and is compiled in the online database Xenbase (Bowes *et al.* 2010; Segerdell *et al.* 2013). Prior experiments have utilized this database in differential expression analyses to identify genes that are specific to certain developmental time points or individual regions of the body at the single-cell level (Briggs *et al.* 2018). In the *X. laevis* brain specifically, RNA expression has been measured in the first 3 days of development to Nieukoop and Faber (NF) stage 44 (Nieuwkoop and Faber 1956; Session *et al.* 2016). However, following this developmental period, transcript expression in the *Xenopus* brain has not yet been thoroughly analyzed, nor has expression in individual regions of the brain (as opposed to a whole-brain approach) been examined.

Metamorphosis is a significant transitional period in amphibian development, marked by drastic thyroid hormone-driven changes in physiology and gene expression across the body, including the central nervous system (Kollros and Frieden 1961; Yaoita and Nakajima 2018). In the amphibians *Microhyla fissipes* (Zhao *et al.* 2016) and *Ambystoma velasci* (Palacios-Martinez *et al.* 2020), transcriptomic profiles over the metamorphic period have been collected and compared to illuminate how gene expression changes as metamorphosis proceeds. In *Xenopus*, gene expression in the regenerating tail and spinal cord has been closely examined during this period as well (Love *et al.* 2011; Edwards-Faret *et al.* 2021). However, the transcriptomic changes that occur over metamorphosis in other regions of the *Xenopus* nervous system have been comparatively understudied. In particular, the visual system’s connections and function in the midbrain have been shown to undergo significant remodeling during metamorphosis in response to thyroxine (Hoskins and Grobstein 1985). Thyroid hormone injection into the developing midbrain between NF stages 46 and 49 has also been shown to increase neural progenitor cell (NPC) proliferation as well as subsequent differentiation (Bestman *et al.* 2012; Bestman *et al.* 2015; Thompson and Cline 2016), indicating a sensitivity to thyroid hormone regulation in the midbrain during this window, but the changes in gene expression underlying this shift have not been well-documented.

*Xenopus* metamorphosis can be subdivided into three stages. The first, premetamorphosis, occurs from NF stages 46 to 53/54 and is marked by low levels of mitosis in the brain (Miyata and Ose 2012; Thuret *et al.* 2015; Moreno and Gonzalez 2017). By this period, the animal has a fully functional nervous system and demonstrates swimming and avoidance behaviors (Dong *et al.* 2009; Shen *et al.* 2011; McKeown *et al.* 2013). Premetamorphosis precedes prometamorphosis (NF stages 53/54–57/58), a period in which neurogenesis dramatically increases (Wen *et al.* 2019). The third stage is the metamorphic climax (NF stages 57/58–66), in which proliferation and neurogenesis fall below even premetamorphic levels (Thuret *et al.* 2015). The metamorphic climax is also significant in that it is thought to parallel many aspects of perinatal mammalian development, including a reorganization of the nervous system accompanying the transition to breathing air and terrestrial life, and a dramatic migration of the eyes to the top of the head resulting in a shift of the visual map in the brain (Nieuwkoop and Faber 1956; Udin and Fisher 1985; Udin 1989; Holzer and Laudet 2013; Yaoita and Nakajima 2018). Given these observations, metamorphosis appears to be an important period in *Xenopus* brain development and elucidating its transcriptional dynamics would help clarify how these significant physiological changes are driven and regulated.

We thus sought to accomplish three goals. First, we set out to expand the existing dataset of *X. laevis* gene expression over brain development and across brain regions, quantifying gene expression at several developmental stages leading to metamorphosis as well as in the fore-, mid-, hindbrain, and spinal cord regions. Secondly, we aimed to utilize these data to gain insight into the cell types and biological processes that are altered over the course of metamorphosis. To assess the drastic changes occurring in the visual system during metamorphosis, we analyzed the midbrain across developmental stages. As changes in neural cell proliferation and differentiation rates have already been shown to occur during this period (Thuret *et al.* 2015), we were particularly interested in exploring how these changes might be regulated at the level of gene expression. We utilized NPC genes in addition to known cell-type markers to investigate relative changes in these cell populations over development. And thirdly, given the similar programs of expression between the tadpole brain at metamorphic climax and the perinatal mouse brain (Yaoita and Nakajima 2018), we examined if similar patterns of expression also exist in the preceding developmental stages in the prometamorphic tadpole and late-prenatal mouse. Utilizing both temporal comparisons across development as well as spatial comparisons across brain regions has helped reveal the cell type and biological pathway dynamics in the developing *Xenopus* brain during metamorphosis. 

## Materials and methods

### Animals

Albino *X.* *laevis* tadpoles of both sexes [RRID: XEP_Xla200] were obtained from an in-house colony or Xenopus Express (Brooksville, FL, USA). Animals were reared in 0.1× Steinberg’s solution at 22°C under a 12 h light/12 h dark cycle, and euthanized with 0.1% MS-222. Animals were staged according to Nieuwkoop and Faber (1956). All animal protocols were approved by the Institutional Animal Care and Use Committee of Scripps Research (approval # 08-0083-3).

### Sample collection and processing

**Figure 1:**
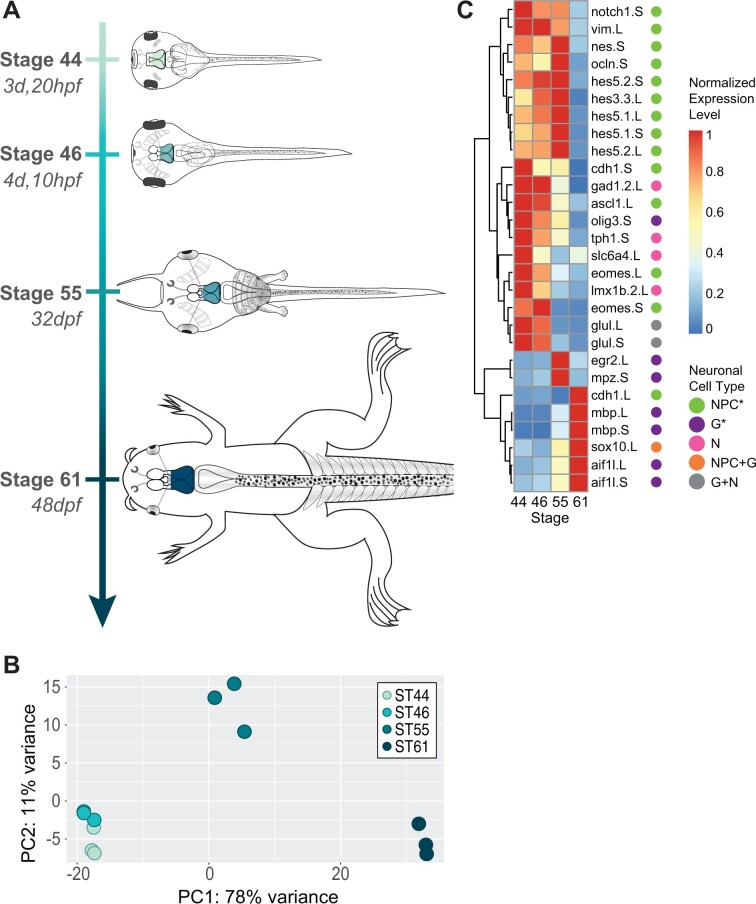
Expression analysis in the *Xenopus* midbrain across developmental stages. (A) *X. laevis* developmental stages sequenced based on Nieuwkoop and Faber (1956) (hpf and dpf indicate hours/days postfertilization). (B) Principal component analysis of samples collected from the *X. laevis* midbrain (MB) at developmental stages 44, 46, 55, and 61. Colors correspond to developmental stages in (A). (C) Heat map of cell-type marker expression over tadpole midbrain development. Cell types are categorized for genes listed as NPC (including neuroepithelial cells, radial glia, intermediate progenitors, and immature neurons); G, glial cell (including mature oligodendrocytes, Schwann cells and Schwann cell precursors, astrocytes, and microglia); N, neuron; NPC + G, progenitor cell/glial marker; G + N, glial/neuron marker. Genes are clustered by similarity in expression across stages with complete linkage clustering, as indicated by the dendrogram to the left. Asterisks indicate significantly enriched cell types (*P* < 0.001 for progenitor cell markers, *P* < 0.005 for glial cell markers). Expression is normalized per gene to the highest expression for that gene.

Three tissue samples were collected from *X. laevis* [RRID: XEP_Xla300] midbrains at the four developmental stages described in Figure  1A: ST44, ST46, ST55, and ST61 (Nieuwkoop and Faber 1956). Three samples were also collected from the subcortical forebrain, midbrain, hindbrain, and spinal cord at ST46 as described in Figure  3A. The hindbrain samples included the rhombic lip. Samples were prepared as described in Huang *et al.* (2021). Three biological replicates were analyzed for each stage and region. Briefly, total RNA was extracted (mirVana, Life Technologies) and samples with an RNA Integrity Number (RIN) > 8 were used for subsequent analysis. A total of 2 ng RNA was amplified to 2–3μg of cDNA (Ovation RNA-Seq System V2, Nugen), purified (Agencourt AMPure XP, Beckman Coulter), and used for library preparation (KAPA Taq PCR kit). PCR products between 200 and 500 bp were selected by gel purification and subjected to single-end 100-bp reads by next-generation sequencing (HiSeq2000, Illumina; RRID: SCR_020132). Samples were multiplexed in one lane at TGEN. Each sample has between 17 and 20 million reads (Supplementary Material S1). Raw and processed counts are available in the GEO repository as GSE183193.

We previously aligned samples collected from animals enriched in either immature neurons or NPCs as detailed in Huang *et al.* (2021) against the *X. laevis* v9.2 genome assembly on Xenbase (Karimi *et al.* 2018) [RRID: SCR_003280] using two read mapping programs, STAR v2.5.2a (Dobin and Gingeras 2015) [RRID: SCR_005622] and HISAT2 v2.0.4 (Kim, Langmead, and Salzberg 2015) [RRID: SCR_015330]. As both aligners yielded comparable results (Huang *et al.* 2021), we chose to proceed with STAR for this dataset. We then counted and assigned the alignments to genes using HTSeq v0.11.1 (Anders, Pyl, and Huber 2015) [RRID: SCR_005514]. Finally, we normalized the counts using the DESeq2 v1.22.2 package (Love, Huber, and Anders 2014) [RRID: SCR_015687] for further analysis.

For the comparison to mouse brain development, we used a published RNA-seq dataset collected from the cerebral cortex (Ctx), specifically the dorsolateral prefrontal cortex, and the cerebellum (Cb) at timepoints from embryonic days 13.5 to 18.5 (PRJEB26869; Cardoso-Moreira *et al.* 2019). We aligned the samples to the Ensembl GRCm38.p6 assembly (Howe *et al.* 2020) and analyzed the data using the same pipeline as we did for *X. laevis*.

### Data analysis and visualization

We also used DESeq2 to perform differential analysis. Unless otherwise specified, we considered genes with both a log2 fold change magnitude of >2 and a Benjamini–Hochberg-corrected FDR <0.05 to be differentially expressed. We used DEGreport v1.18.1 (Pantano 2019) [RRID: SCR_018941] with default settings for cluster analysis of differential expression analysis results. We used GOseq v1.34.1 (Young *et al.* 2010) [RRID: SCR_017052] to identify GO-term enrichment in sets of differentially expressed genes. To determine gene list enrichment, we used a two-sided Fisher’s exact test and a cutoff of *P* = 0.05 over a background set of all detected genes. We used the R package pheatmap v1.0.12 (Kolde 2019) [RRID: SCR_016418] to generate the heatmaps.

### Interspecies gene conversion

To make comparisons between *Xenopus* and Mouse transcriptomes, we chose to bridge the gap by converting both datasets to their human homologues. To address the incomplete *Xenopus* gene ontology (GO) annotation despite the existence of annotated *Homo* *sapiens* sequence homologs, Briggs *et al.* (2018) previously mapped *Xenopus tropicalis* gene symbols to the *H. sapiens* genome. We further extended this mapping to connect the closely related *X.* *laevis*, whose GO annotation is similarly lacking, to the *H. sapiens* genome. To identify homologous genes between *X. laevis* and *H. sapiens*, we first aligned *X. laevis* gene symbols using BLAST to the closely related *X. tropicalis* genome. This table was then used to map the *X. laevis* gene symbols to *H. sapiens*, with *X. tropicalis* as an intermediary (Supplementary Material S2). Of the 91,611 *X. laevis* transcripts, 90.77% (83,159) were mapped to the *X. tropicalis* transcriptome with high confidence. A total of 70.23% (64,399) of the *X. laevis* transcripts were ultimately mapped to *H. sapiens* gene symbols. We used biomaRt v2.38.0 (Kinsella *et al.* 2011) [RRID: SCR_002987] to identify homologous genes between *Mus* *musculus* and *H. sapiens*, as a bridge between the mouse and tadpole gene lists.

## Results

### Changes in gene expression in the developing tadpole midbrain

During the development, *Xenopus* tadpoles undergo drastic physiological changes leading up to metamorphosis (Nieuwkoop and Faber 1956; Nishikawa and Hayashi 1994; Pronych and Wassersug 1994; Figure  1A), which are coupled to significant changes in gene transcription (Thuret *et al.* 2015; Yaoita and Nakajima 2018), yet little is known about the broad transcriptional changes occurring in the developing brain during this time. In particular, the midbrain, which includes the optic tectum, the central visual processing center in amphibians, undergoes considerable changes with consequences in electrophysiological and behavioral responses (Akerman and Cline 2006; Dong *et al.* 2009). To investigate the developmental changes in gene transcription in this region, we isolated and sequenced RNA from the midbrains at NF stages 44, 46, 55, and 61 (Nieuwkoop and Faber 1956) and compared the samples through differential expression analysis (Figure  1). Principal component analysis of these midbrain samples showed that they clustered by developmental stage (Figure  1B), indicating that changes in gene transcription by stage were consistent across different clutches of animals. In total, we identified 3358 genes that were differentially expressed between at least two stages. The analysis also revealed that samples taken from stage 44 and 46 were similar in expression to one another. In fact, only four genes were differentially expressed between these two stages, about 10-fold less than any other pairwise analysis (Supplementary Material S3). In comparison, there were 799 genes differentially expressed comparing stages 44 and 55; 724 genes comparing stages 46 and 55; 2319 genes comparing stages 44 and 61; 2358 genes comparing stages 46 and 61; and 1009 genes comparing stages 55 and 61 (Supplementary Material S3).

Using these differential expression datasets, we first examined how other known cell-type markers are expressed in the midbrain over time. Using a list of canonical neural cell-type markers (Supplementary Material S4), we tested for enrichment over the stages of metamorphosis (Figure  1C). We found that glial cell markers (including mature oligodendrocytes, Schwann cells and Schwann cell precursors, astrocytes, and microglia) were enriched in stages 55 (*P* = 0.003) and 61 (*P* = 0.008) compared to the earlier two stages, while progenitor cell markers (including neuroepithelial cells, radial glia, intermediate progenitors, and immature neurons) were enriched in stages 44, 46, and 55 compared to stage 61 (*P* = 0.0006). Specifically, radial glia markers including *hes5* (*P* = 0.001) and immature neuron markers including *eomes* (*P* = 0.008) were enriched in these three earlier stages compared to the latest stage. These data are consistent with a developmental progression from a more proliferative state to a more differentiated state in the tadpole brain (Yaoita and Nakajima 2018).

We also investigated how relative populations of NPCs and immature neurons changed in the tadpole midbrain over development. We used the NPC- and immature neuron-associated gene lists obtained in Huang *et al.* (2021) as indicators of a transition from a timepoint enriched in one cell type to a timepoint enriched in the other. Testing for enrichment, we found that immature neuron-associated genes were significantly depleted over genes that were downregulated in stages 44 and 46 and were upregulated at stage 61 (*P* < 0.0001). Examining specific genes in this set, we observed downregulation of known neural differentiation regulators such as *wnt1* (Kondo *et al.* 2011) and *foxg1* (Dastidar *et al.* 2011) in stage 61 compared to earlier stages (Supplementary Material S3) again suggesting broad changes in immature neuron populations over this period.

**Figure 2:**
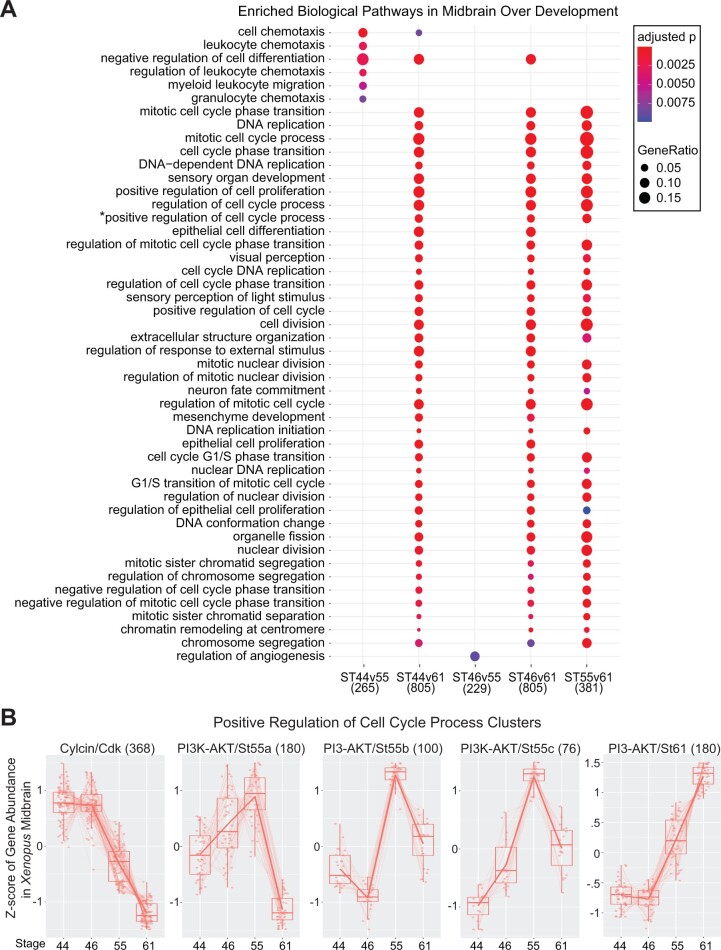
Enriched biological pathways in the *Xenopus* midbrain across developmental stages. (A) GO biological pathway analysis showing enriched sets of genes differentially expressed between stages of tadpole midbrain development. Dot size corresponds to the proportion of differentially expressed genes associated with the biological pathway, and dot color refers to the adjusted *P*-value. The numbers along the *x*-axis correspond to the total number of genes differentially expressed in each comparison. (B) Clusters of genes differentially expressed during tadpole midbrain development that are associated with the “Positive Regulation of Cell Proliferation” GO category. The five clusters shown are Cyclin/Cdks (368 genes), PI3K-AKT/St55a-c, and PI3K/St61, numbers in parentheses indicates the number of genes in each cluster. The Cyclin/Cdk cluster is enriched in cell cycle-related genes at stages 44–46 (*P* < 0.001) and the remaining clusters are enriched in PI3K-AKT pathway associated genes (St55a-c, *P* = 0.019; St61, *P* < 0.001).

To investigate the processes involved in midbrain development, we performed GO analysis on our differentially expressed genes across stages (Supplementary Material S5). As noted previously, we observed few differentially expressed transcripts between stages 44 and 46, and as such did not explore further their enriched GO terms. However, comparisons against stages 55 and 61 revealed distinct genes and enriched biological functions in each (Figure  2). This was especially visible in the stage 44/55 and the stage 46/55 comparisons, which despite possessing a similar number of overall genes (265 and 229, respectively), displayed incongruous enriched functions. This suggests that differences in midbrain expression between stages 44 and 46 may possess biological significance that we were unable to elucidate at the bulk-tissue level with our number of samples. Overall, we found that genes that were differentially expressed between two or more stages were enriched for GO terms pertaining to cell division, development, and cell cycle transitions (Figure  2A), suggesting broad changes in the regulation of these processes over this period of midbrain development. In particular, genes involved in positive regulation of cell proliferation as well as negative regulation of cell differentiation were highly enriched across comparisons between the first three stages and stage 61. We noted that similar functions were enriched in each of these comparisons (Figure  2A; ST44v61, ST46v61, ST55v61). Thus, we wished to more closely examine the specific genes involved at each timepoint in one GO term to identify stage-specific subgroups. To further investigate these patterns of gene expression in the midbrain over development, we performed cluster analysis on genes identified as being involved in the “positive regulation of proliferation” GO category and identified five significant expression pattern clusters containing 76–368 genes each (Figure  2B). We uncovered three distinct groups with stage-specific expression peaks: one was increased at stages 44/46 with 368 genes, three at stage 55 with 180, 100, and 76 genes each, and one at stage 61 with 180 genes (Figure  2B; Supplementary Material S6). The cluster with elevated expression at the early stages was highly enriched in cell cycle regulation genes, including a number of cyclins and cyclin-dependent kinases (Cyclin/Cdk Cluster). The remaining 4 clusters, groups with elevated expression at stages 55 or 61, were all enriched in genes involved in the PI3K-AKT signaling pathway (PI3K-AKT/St55a-c, PI3K-AKT/St61; Figure  2B). In addition to broad processes such as proliferation being differentially expressed over midbrain development, more specific pathways like PI3K-AKT also showed temporal enrichment at the latter two developmental stages, despite an expected decline in proliferation by stage 61 (Yaoita and Nakajima 2018). While we observed an overall decrease in NPC-related transcripts over time from our cell-type marker analyses, examining process-specific expression revealed a more nuanced pattern of expression dynamics.

### Differences in regional gene expression at NF stage 46

**Figure 3:**
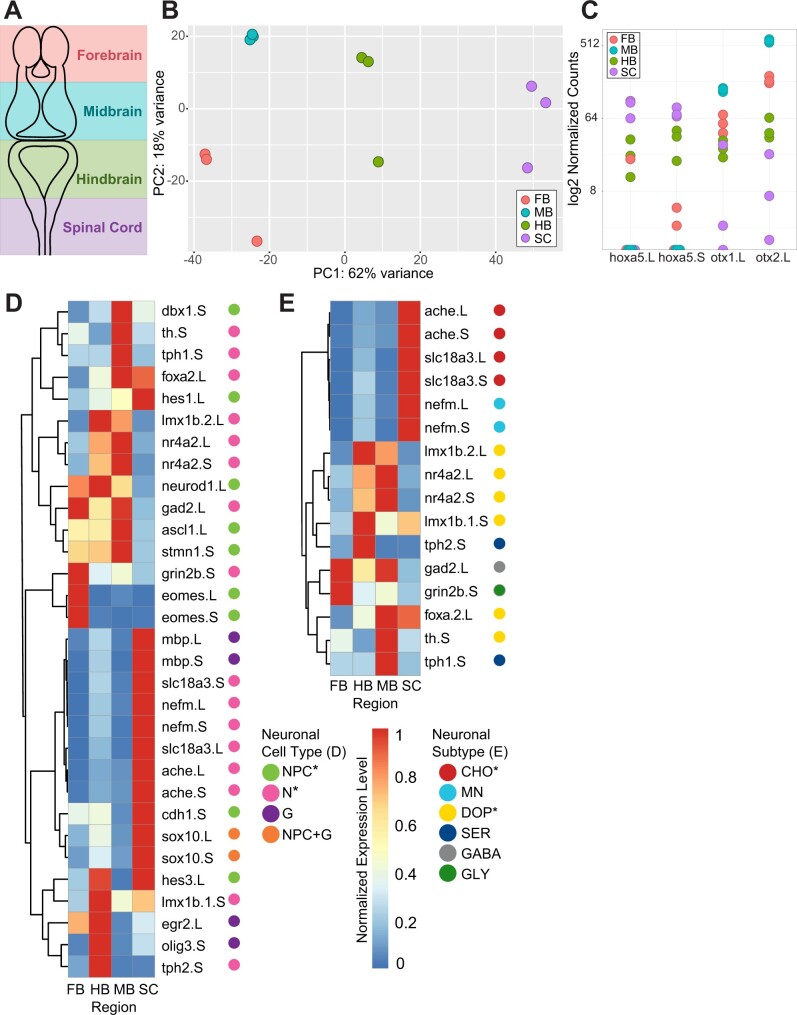
Expression analysis of *Xenopus* stage 46 across different brain regions. (A) Schematic depicting the four brain regions dissected and sequenced at *Xenopus* Stage 46: forebrain (pink), midbrain (blue), hindbrain (green), and spinal cord (purple). (B) Principal component analysis of samples collected from the *X. laevis* stage 46 brain in the forebrain (FB ST46, pink), midbrain (MB ST46, blue), hindbrain (HB ST46, green), and spinal cord (SC ST46, purple). Colors correspond to brain regions in (A). (C) Normalized expression of *hox5*, a hindbrain-spinal cord marker, and *otx1* and *otx2*, forebrain–midbrain markers, across all brain regions. Colors correspond to brain regions in (A, B). (D) Heat map of cell-type marker expression over ST46 tadpole brain regions. General cell types are categorized for genes listed as NPC (including neuroepithelial cells, radial glia, intermediate progenitors, and immature neurons); G, glial cell (including mature oligodendrocytes, Schwann cells and Schwann cell precursors, astrocytes, and microglia); N, neuron; NPC+G, progenitor cell/glial marker. (E) Heat map of neuron subtype marker expression over brain region at stage 46. Specific neuronal subtypes are categorized for genes listed as CHO, cholinergic neuron; MN, mature neuron; DOP, dopaminergic neuron; SER, serotonergic neuron; GABA, GABAergic neuron; GLY, glycinergic neuron. Genes are clustered by similarity in expression across regions with complete linkage clustering, as indicated by the dendrogram to the left. Asterisks indicate significantly enriched cell types (*P* < 0.002 for PG, *P* < 0.001 for N, *P* < 0.001 for both DOP and CHO markers). Expression is normalized per-gene to the highest expression for that gene.

During premetamorphosis, a progenitor quiescent period has been observed in the tadpole pallium in the dorsal region of the forebrain (Moreno and Gonzalez 2017) as well as in the posterior hindbrain (Thuret *et al.* 2015). However, a comparison of gene expression to the midbrain during this period as well as comparisons between the forebrain, hindbrain, and spinal cord have not yet been carried out. To investigate the gene expression patterns across brain regions at this stage, we performed differential expression analysis between RNA samples harvested at NF stage 46 from the fore-, mid-, hindbrain, and spinal cord (Figure  3A). Principal component analysis showed clear clustering by brain region along the primary axis, confirming precision of tissue isolation (Figure  3B); some intra-region variance is present, but to a much lesser extent than the inter-region difference. Using these samples, we identified all genes that were differentially expressed between one brain region and all other brain regions. From 2974 genes that were differentially expressed between at least two stages 46 brain regions, we found that 138 genes were specifically differentially expressed in the forebrain compared to all other regions; 301 in the midbrain; 45 in the hindbrain; 279 in the spinal cord (Supplementary Material S7). As predicted, anterior–posterior patterning genes were present in the expected brain regions : *otx* genes in the forebrain and midbrain, and *hox* genes in the hindbrain and spinal cord (Schilling and Knight, 2001; Figure  3C). We also noted the presence of *hoxa5.L* in one forebrain sample as well as otx in most spinal cord samples. However, given that expression in these samples was highly varied as well as lower than in the other brain regions, the expected pattern of regional expression remains clear.

Differential expression analysis across stage 46 brain regions revealed that of genes upregulated in the midbrain, NPC-associated genes were depleted in the midbrain–forebrain comparison (*P* = 0.0282) as well as the midbrain–hindbrain comparison (*P* = 0.0058), but not the midbrain–spinal cord comparison (Supplementary Material S7). Furthermore, using the list of known cell markers, we found that progenitor markers (*P* = 0.002) and neuronal markers (*P* < 0.0001) were enriched amongst genes that were differentially expressed across regions (Figure  3D). Specifically, progenitor/immature neuron markers like *eomes* were downregulated in the spinal cord compared to the other, more proliferative, brain regions (*P* < 0.0001). We next looked at the differential expression of specific neuronal cell-type markers across brain regions and found that dopaminergic (DOP) neuron markers were enriched in the midbrain and hindbrain, while the cholinergic (CHO) neuron markers *ache* and *slc18a3* were enriched in the hindbrain and spinal cord (*P* < 0.0001; Figure  3E). The latter observation may be due to the significant presence of cholinoceptive cells in the hindbrain and spinal cord (Thiriet *et al.* 1992), while the former is consistent with where the majority of DOP neurons are known to reside (Blum 1998; Hegarty *et al.* 2013). These data indicate that neuronal subtype populations express transcripts at a sufficient level to be identified in bulk-tissue analysis. Meanwhile, NPC populations in the forebrain, midbrain, and hindbrain, though not the spinal cord, appear to be comparable during premetamorphosis.

**Figure 4:**
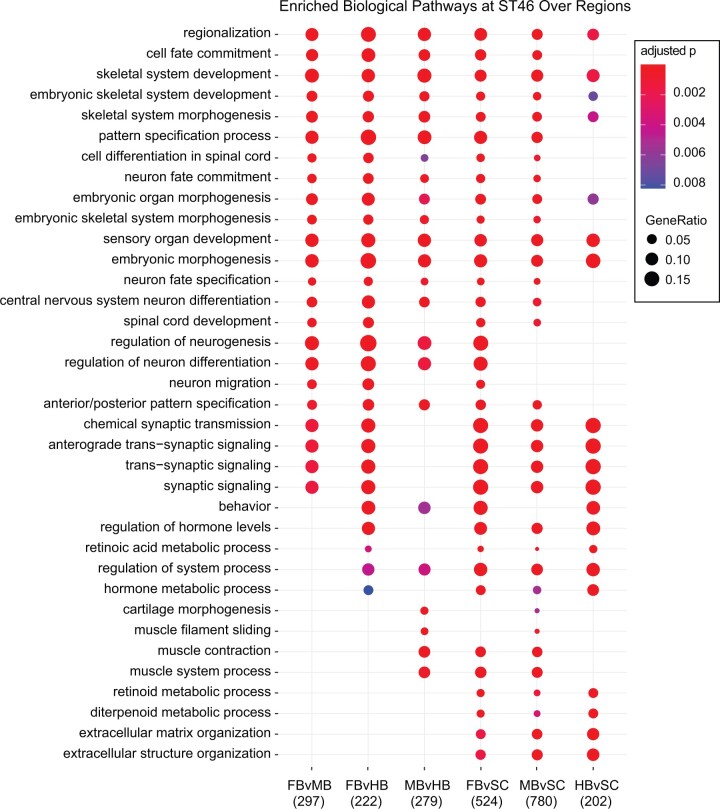
Enriched biological pathways in ST46 *Xenopus* across brain regions. GO biological pathways enriched in the set of genes differentially expressed between two regions of the tadpole stage 46 brain. Brain regions, as shown in Figure  3A are FB, forebrain; MB, midbrain; HB, hindbrain; and SC, spinal cord. Dot size corresponds to the proportion of differentially expressed genes associated with the biological pathway, and dot color refers to the adjusted *P*-value. The numbers along the *x*-axis correspond to the total number of genes differentially expressed in each comparison.

To investigate the patterns of gene expression changes across different brain regions, we performed GO analyses of pairwise comparisons between regions (Supplementary Material S8). As expected, across all regions we found significant enrichment in GO terms pertaining to regional development and pattern specification, though the comparison between the hindbrain and spinal cord resulted in fewer significant terms (Figure  4). The lack of enrichment in this comparison indicates transcriptional similarity between the two regions in regard to pattern specification. Though neuron fate commitment and differentiation were also enriched in many pairwise comparisons, investigating the tagged genes revealed that many of them were also regionalization related. For instance, we found *gsx2*, a known regulator of telencephalon progenitor maturation (Pei *et al.* 2011), to be highly expressed in the forebrain compared to all other regions. Similarly, we observed *pitx2*, *lef1*, and *tcf7l2* specifically in the midbrain. These wnt-mediating genes have also been observed primarily in mesodiencephalic DOP neurons (Martin *et al.* 2004; Nouri *et al.* 2020), consistent with our observation of DOP neuron marker enrichment in the midbrain (Figure  3E). Thus, pathway enrichment in the regional comparisons also appear to reflect regional differences rather than any significant disparities in proliferation or differentiation.

### Comparing regional expression between *X. laevis* and *M. musculus*

Previous studies have shown that similar genes are upregulated in both the postnatal rodent brain and in the *Xenopus* brain during metamorphosis (Yaoita and Nakajima 2018). Similar patterns of thyroid hormone-associated gene expression have been observed in both species during the first three postnatal weeks in mice and the metamorphic climax (NF stages 57/58–stage 66) in *Xenopus* (Miyata and Ose 2012; Holzer and Laudet 2013; Yaoita and Nakajima 2018). Given this, we sought to examine if this interspecies parallel extends earlier into the developmental process.

**Figure 5:**
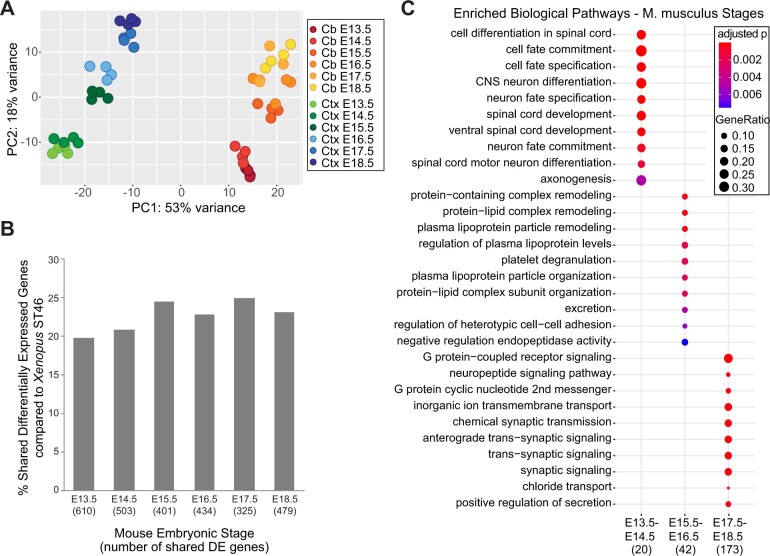
Expression analysis of mouse brain regions across development. (A) Principal component analysis of mouse Ctx and Cb data, collected at timepoints E13.5–E18.5. (B) Proportion of genes differentially expressed in the mouse Ctx *vs* Cb at each timepoint that was also differentially expressed in the tadpole stage 46 forebrain *vs* hindbrain. The total number of differentially expressed genes in at this timepoint is indicated in parentheses next to the timepoint on the *x*-axis. All timepoints in the mouse model shared a similar proportion of genes with the tadpole timepoint. (C) GO biological pathways enriched in the set of genes upregulated in both the mouse Ctx and Cb at developmental timepoints from E13.5 to E18.5. Distinct pathways are upregulated at each developmental period. Dot size corresponds to the proportion of differentially expressed genes associated with the biological pathway, and dot color refers to the adjusted *P*-value. The numbers along the *x*-axis correspond to the total number of genes differentially expressed in each comparison.

First, to identify changes in gene expression across mouse brain development, we compared samples between the *M. musculus* cortex (Ctx) and cerebellum (Cb) at the six developmental stages between E13.5 and E18.5 (PRJEB26869; Cardoso-Moreira *et al.* 2019). PCA analysis showed expected clustering by developmental stage in both regions (Figure  5A). Comparing the mouse Ctx and Cb at each timepoint, we found that differentially expressed genes were highly conserved at each timepoint. Hundred and eighty-three genes were differentially expressed between the two regions at all timepoints, composing a robust set of genes that varied in expression regionally but not temporally (*P* < 0.0001).

Examining homologous genes in *X. laevis*, we found that about a third of the genes differentially expressed between the mouse Ctx and Cb at all timepoints were also differentially expressed between the *Xenopus* forebrain and hindbrain at stage 46 (*P* < 0.0001; Supplementary Material S9). These genes consisted primarily of homeobox and transcriptional regulation genes, and as such were significantly enriched in GO terms pertaining to regionalization and pattern specification. This is consistent with the anterior to posterior regionalization in both the mouse cortex and Cb samples as well as the forebrain and hindbrain tadpole samples, despite the amphibian forebrain samples not including cortical tissue. For instance, we consistently observed *irx*-family genes (IRX3-6), already known to be involved in patterning and anteriorly bound in expression at the prethalamus (Rodriguez-Seguel *et al.* 2009), as being expressed highly in the mouse Cb compared to the Ctx at all stages E13.5–E18.5, as well as in the tadpole hindbrain compared to the forebrain at stage 46. As there was not a significant difference in the proportion of genes shared between any particular timepoint in the mouse model and the tadpole model at stage 46 (Figure  5B), these homeobox genes seem to possess a region-specific pattern of expression that is conserved both across species as well as development. Excluding these invariable genes, we then identified three additional homeobox genes that were differentially expressed between the tadpole stage 46 forebrain and hindbrain as well as between the Ctx and Cb at a single developmental stage in mouse: *hoxb5* at E17.5, *hoxe4* at E18.5, and *hoxd4* at E18.5. Unlike other homeobox genes, these genes showed differential expression between the mouse brain regions only in the later stages observed; they also showed similar expression in the tadpole at stage 46. Overall, we found that pattern specification genes were broadly conserved across both species during the observed time periods.

### Comparing temporal expression between *X. laevis* and *M. musculus*

Having established that region-specific expression is significant and largely conserved across development, we next sought to examine temporal expression within each region. Separately in the mouse Ctx and Cb, we identified genes that were upregulated at each of the embryonic stages E13.5/E14.5, E15.5/E16.5, and E17.5/E18.5 compared to the other timepoints. We chose to consider genes that showed coincident expression in both regions, displaying higher expression in both the Ctx and Cb at each timepoint. By requiring the genes to have similar expression in both, we aimed to curtail region-specific genes and obtain a more general picture of temporal expression across the brain. From 3585 total differentially expressed genes in the Ctx and 2419 in the Cb, 1092 were upregulated in both regions at the same timepoints (Supplementary Material S10). We found that genes upregulated at each timepoint displayed enrichment in distinct GO biological pathways (Supplementary Material S11). Fate commitment and differentiation were enriched at E13.5/E14.5; lipoproteins including *Apoa1* and *Apob* were enriched at E15.5/E16.5; synaptic signaling was enriched at E17.5/E18.5 (Figure  5C). Taken together, these timepoints demonstrate a sequential pattern of pathway enrichment shared between both the mouse Ctx and Cb.

**Figure 6:**
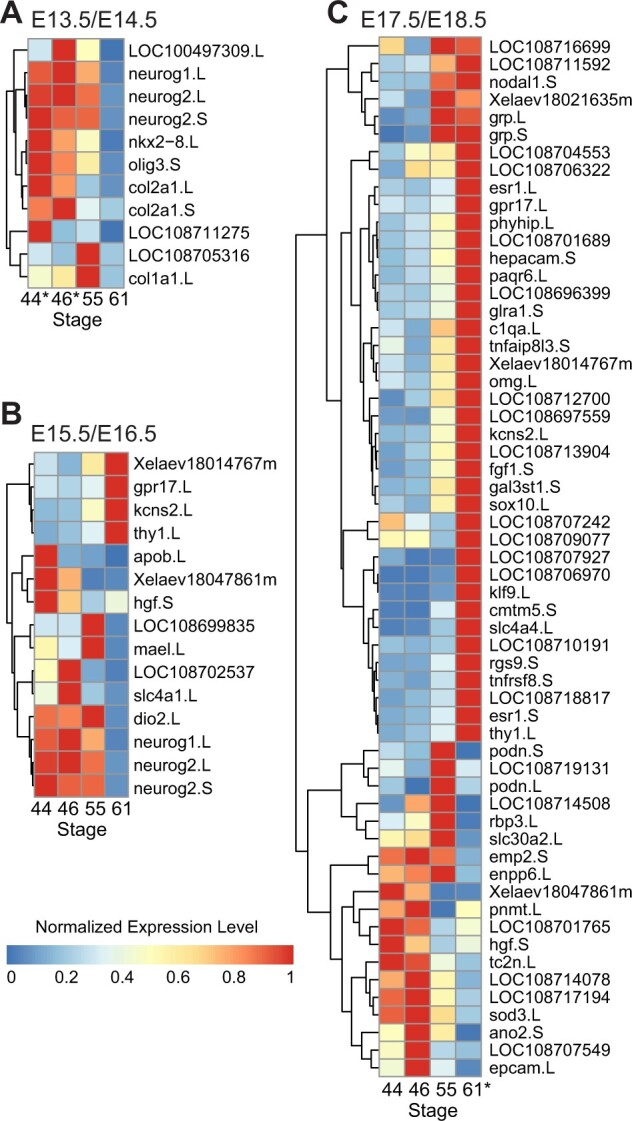
Comparison of gene expression between *Xenopus* and Mouse across development. (A) Heat map showing expression of genes upregulated in the mouse Ctx and Cb at E13.5/14.5 over the ST44-61 *Xenopus* midbrain. (B) Heat map showing expression of genes upregulated in mouse Ctx and Cb at E15.5/E16.5 over the ST44-61 *Xenopus* midbrain. (C) Heat map showing expression of genes upregulated in the mouse Ctx and Cb at E17.5/18.5 over the ST44-61 *Xenopus* midbrain. Genes are clustered by similarity in expression across *Xenopus* midbrain stages with complete linkage clustering, as indicated by the dendrograms to the left. E13.5/E14.5 genes were overrepresented in genes upregulated at *Xenopus* ST44 and ST46 (*P* < 0.0001), while E17.5/E18.5 genes were overrepresented in genes upregulated at stage *Xenopus* ST61 (*P* < 0.0001). Expression is normalized per gene to the highest expression for gene.

Having obtained a nonspatial, temporal-specific profile of gene expression in the mouse brain, we next wanted to compare expression in the developing *Xenopus* midbrain to see if a similar timeline of pathway enrichment existed between mouse and tadpoles during development. Examining these genes over the tadpole midbrain from stages 44 to 61, we found that many of them displayed a similar pattern of temporal expression (Figure  6). Genes upregulated at E13.5/E14.5 in the mouse model significantly tended to also be upregulated at stages 44 and 46 in the tadpole midbrain (*P* = 0.0001; Figure  6A), while those upregulated at E17.5/E18.5 were significantly upregulated at stage 61 (*P* < 0.0001; Figure  6C). The intermediate E15.5/E16.5 genes were somewhat biased toward stages 44 and 46 (*P* = 0.04; Figure  6B), but also had a fair number of genes highly expressed at the later timepoints as well. Overall, the expression of the genes upregulated at each timepoint in the mouse brain followed a similar sequence of expression in the *Xenopus* midbrain. These data suggest that patterns of gene expression in the brain across development are conserved, at least in part, across species.

## Discussion

To investigate the changes in gene transcription during *X. laevis* development, we conducted differential expression analyses of the developing tadpole brain. We analyzed expression over stages of development in the midbrain, and across regions of the brain at a single developmental stage. We found that genes involved in positive regulation of NPC proliferation as well as known progenitor cell markers were upregulated in the midbrain prior to metamorphic climax, and that at the start of metamorphosis, neural progenitor populations appeared to be similar across the fore-, mid-, and hindbrain regions. Lastly, we compared gene expression patterns between early tadpole metamorphosis and mid- to late-embryonic mouse development and found that regulation of biological processes like cell fate commitment and synaptic signaling follow similar trajectories in the brain across species.

### Gene expression over *X. laevis* midbrain development

We observed a sharp decrease in radial glial and immature neuron markers at stage 61 in the tadpole midbrain compared to the three earlier stages. Prior studies have shown that both neuronal birth and neural progenitor proliferation decrease dramatically between stages 56 and 66 in the posterior hindbrain (Thuret *et al.* 2015) following prometamorphosis, as well as by stage 66 in the optic tectum (D'Amico *et al.* 2013). As such, this observation seems in line with these previous findings. However, we did not clearly observe a similarly reported secondary wave of neurogenesis at the onset of prometamorphosis, between stages 54 and 56. Though Stage 55 samples displayed the highest expression of many radial glia and immature neuron markers, this difference was not significant compared to stages 44 and 46. As proliferation activity decreases from its observed peak at stage 54 in the hindbrain to premetamorphosis levels by stage 56 (Thuret *et al.* 2015), it is possible that this decrease is more abrupt than previously suspected. Proliferation in the midbrain may also express an early-shifted pattern of activity compared to the hindbrain, peaking and returning to premetamorphic levels within the stage 46 to stage 54 window. Collection of additional data at timepoints within this window across the different brain regions would allow investigation of both possibilities.

We also observed that myelination-related glial marker genes such as *mbp* (Roach *et al.* 1985) were enriched at the later stages compared to earlier ones (Figure  1C); this included genes normally associated with myelinating Schwann cells in mammalian models, such as *egr2* and *mpz* (Lemke and Axel 1985; Zorick *et al.* 1996). However, *mpz* has also been found to be expressed in the central as well as the peripheral nervous systems of some species such as trout (Brosamle and Halpern 2002). In zebrafish, it has been shown to even be absent from Schwann cells entirely (Brosamle and Halpern 2002), but present in the optic tectum starting at 4 weeks postfertilization and upregulated following optic nerve crush (Schweitzer *et al.* 2003). It is possible that *mpz* may play a corresponding role in *Xenopus* as in zebrafish in central nervous system myelination. Similarly, *egr2* and other mammalian Schwann cell-associated genes may also be differentially specific in amphibian models.

Using our cell-type-associated gene lists, we observed significant depletion of immature neuron-associated genes in the set of genes downregulated at stages 44 and 46, as well as in the set upregulated at stage 61. It is likely that changes in expression pertaining to cell-type enrichment are somewhat obscured by other developmental changes; we observed no enrichment of the NPC-associated genes over development. However, this depletion does fall in line with our previous observations using marker genes, in that it suggests an increase in the relative immature neuron population does not occur between these timepoints. Future studies comparing less dynamic conditions than developmental timepoints may be able to leverage these progenitor- and immature neuron-associated genes with greater resolution.

Examining the expression of proliferation-promoting genes across midbrain development, we found that cyclins and cyclin-dependent kinases decreased in expression over time. For instance, we found cyclin D1 and cyclin-dependent kinase 2 to be significantly downregulated following prometamorphosis. Such genes are critical for the timing of progression through the cell cycle (Vernon and Philpott 2003). In mice, their inhibition has been shown to lead to a lengthening of the G1 phase and a decrease in proliferative division (Calegari and Huttner 2003). This again agrees with the expected decline in proliferation after prometamorphosis. Previous studies have also found that the average cell cycle length in the *Xenopus* brain increases during this developmental period alongside proliferation (Thuret *et al.* 2015). This is in contrast to the primate brain, in which cell cycle length begins decreasing partway through cortical neurogenesis (Kornack and Rakic 1998). Cyclin-dependent kinase 2 and other cell cycle regulators have also been shown to be thyroid hormone-regulated in stage 54 *Xenopus* brain (Wen *et al.* 2019). Transcription of thyroid hormone receptors has previously been shown to increase over the course of metamorphosis; for instance *thrb* expression rises dramatically from prometamorphosis at stage 55 to metamorphic climax at stage 61 (Yaoita and Brown 1990), an increase we also observed in our own analysis (*P* < 0.01). Taken together, this increase in thyroid hormone receptor expression and downregulation of cyclin D1 and other G1 phase regulators in tandem with a decrease in neural progenitor markers suggests that cell cycle length and neuronal proliferation in *Xenopus* may share a similar thyroid hormone-mediated mechanistic relationship as in mice.

We also observed that many genes involved in the PI3K-AKT signaling pathway, a positive regulator of proliferation (Roberts *et al.* 2002; Vander Haar *et al.* 2007; Cheng *et al.* 2018) were upregulated in later stages at either stage 55 or 61. This included genes such as *fgf1* and *rictor*. *fgf1* has been shown to play a role in the maintenance and proliferation of neural stem cells (Kalyani *et al.* 1999). As part of mTORC2 (Jacinto *et al.* 2004), knockdown of *rictor* has been shown inhibit dendrite formation in embryonic rat neurons (Skalecka *et al.* 2016). A partial explanation for the discrepancy between the expected decrease in proliferation and the observed mRNA expression of proliferation-upregulating genes could be a concurrent change in posttranscriptional regulation. Studies in the closely related anuran *M.* *fissipes* found that during metamorphosis, miRNAs targeting the PI3K-AKT pathway were the most significantly enriched group, indicating that miRNA regulation plays a significant role in metamorphic development (Liu *et al.* 2018). miRNA expression during *Xenopus* metamorphosis has previously been profiled (Hikosaka *et al.* 2007), but target genes for these miRNAs have yet to be identified. Characterization of these differentially expressed miRNAs could reveal a similar increase in PI3K-AKT pathway targeting in *Xenopus* and underline the importance of posttranscriptional regulation in developmental processes. The PI3K-AKT signaling pathway has also been demonstrated to be a non-genomic thyroid hormone-activated facilitator of neuronal survival (Cao *et al.* 2009). Given this, it is also possible that this increase in PI3K-AKT expression is a consequence of downregulated apoptosis following the increase in thyroid hormone signaling and receptor expression observed during this period (Yaoita and Brown 1990).

### Gene expression across *X. laevis* brain regions

Our brain region samples showed some intra-region variance, which could be reduced with a larger sample size; however, this difference is much smaller than the difference between regions. A larger sample size would also help clarify the presence of pattern specification genes in unexpected regions, such as the presence of *hoxa5.L* in one forebrain sample. As expected, many genes that were differentially expressed between brain regions at stage 46 pertained to pattern specification. In particular, negative regulators of differentiation were highly expressed in the spinal cord compared to other regions. Furthermore, the spinal cord showed significantly reduced expression of intermediate progenitor markers like *ascl1* at this timepoint. While *Xenopus* tadpoles are usually able to regenerate spinal cord injuries prior to adulthood, it is known that they experience a refractory period between stages 45 and 47 in which this ability is briefly lost (Beck *et al.* 2003). Previous studies have found that following tail amputation in non-refractory period tadpoles, proliferation-regulating pathways such as Wnt and BMP become active in the regenerating area (Lin and Slack 2008); significantly lower activity of such pathways is observed in refractory period individuals, indicating that inhibition of cell proliferation likely plays a role in this phenomenon (Kakebeen and Wills 2019). Our observations suggest that differentiation-inhibiting activity may also be already present in the uninjured spinal cord during this period independent of injury, which may contribute to the transient loss of regenerative ability during this period. Further investigation of gene expression in the pre and postrefractory period spinal cord would help illuminate if this inhibition is temporally specific to this timeframe in addition to being regionally specific.

Examining known cell-type markers, we observed depletion of NPC-associated genes in genes differentially expressed in the stage 46 midbrain compared to the forebrain and hindbrain (Figure  4C). In our GO analysis of regional comparisons, though we did see enrichment of genes pertaining to neuronal differentiation and fate commitment (Figure  5), many of these genes were also region-specific and played significant roles in pattern specification, which was to be expected comparing brain regions. Taken together, this may suggest that the relative population of NPCs at this timepoint is similar across all three regions. It has previously been shown that from stage 52/53 to 54/55 at the tail end of premetamorphosis, NPC populations are similar in the *X. laevis* telencephalon, diencephalon, tectum, and Cb (Denver *et al.* 2009). In addition, we demonstrated that neural progenitor proliferation gene activity does not appear to change significantly in the midbrain from stages 44 to 55 (Figure  3D), and that a period of quiescence precedes a secondary wave of neurogenesis and decreased proliferation in the *Xenopus* hindbrain during prometamorphosis (Thuret *et al.* 2015). As stage 46 constitutes the start of premetamorphosis, it is plausible that neural progenitor populations remain similar both across all three brain regions as well as developmental timepoints during this period. As previously mentioned, there remains the possibility that a sharp spike in proliferation may occur at NF stage 54 or earlier, which we would not be able to detect with our current data. Additional intermediate timepoints would be necessary to investigate this further.

### Comparing gene expression between *X. laevis* and *M. musculus*

We found that the dominant biological processes over brain development during E13.5–E18.5 in mouse and NF stages 44–61 in *Xenopus* were generally similar. In both timeframes, the expression of neuron cell fate commitment and differentiation-related genes decreased consistently with each consecutive developmental stage. Meanwhile, synaptic signaling was highest at stage 61 in tadpoles and at the last timepoints in mice; this pattern has previously been observed comparing *in situ* hybridization data in embryonic mice (Thompson *et al.* 2014). This is not to say that the two periods parallel one another in development, but rather that they appear to possess similar patterns of overall expression regarding their major enriched pathways. In fact, enrichment of E15.5/E16.5 mouse genes in the tadpole stage 44/46 midbrain rather than the intermediate stage 55 suggests that this span of mouse brain development may more closely resemble an earlier window of tadpole development.

Regarding differences in regional expression between the species, we observed a high proportion of genes that were differentially expressed in both tadpole and mouse. Considering the robust conservation of homeobox genes across species (Santini *et al.* 2003), this was expected. The only homeobox genes that were not consistently differentially expressed between the mouse Ctx and Cb at all time points were *hoxb5*, *hoxc4*, and *hoxd4*. All are associated with hindbrain expression in the brain (Sjostedt *et al.* 2020), and *hoxc4* and *hoxd4* have additionally been shown to continue increasing in hindbrain expression during the first postnatal stages in mice (Sunkin *et al.* 2013). Because these genes were only differentially expressed between the Ctx and Cb at E17.5 and E18.5 in addition to the tadpole midbrain at stage 46, it is possible that regional development of the premetamorphic tadpole brain at this time is broadly similar to that of the late prenatal mouse. This would support the previous notion that the analyzed window of mouse development is in fact more similar to an earlier period of tadpole development. Further studies experimentally validating this observed differential expression and tracing its downstream effects will help illuminate the extent of this comparability between models.

## Data availability

All raw *X. laevis* data and processed counts are available in the GEO repository as [GSE183193]. All data are available as supplemental files on the GSA figshare portal: https://doi.org/10.25387/g3.16611838. The aligned read statistics are provided in the supplemental material as Supplementary Material S1. The table constructed for conversion of gene symbols from *X. laevis* to *H. sapiens* is provided as Supplementary Material S2. The full differential expression analysis results for the by-stage comparison in the midbrain is provided in Supplementary Material S3; for the by-region comparison at stage 46 in Supplementary Material S7. Full GO term analysis results for these comparisons are provided in Supplementary Material S5 for the by-stage comparison and Supplementary Material S8 for the by-region comparison. The list of cell-type markers used to test for enrichment in the by-stage comparison are given in Supplementary Material S4; the genes annotated as positively regulating proliferation in the same comparison are listed in Supplementary Material S6. The differential expression analysis in mouse comparing the cortex and Cb, and the analysis comparing stages within each region, are provided in Supplementary Material S9 and Supplementary Material S10, respectively. The full GO term analysis comparing these genes in *X. laevis* is given in Supplementary Material S11.

Supplementary material is available at figshare DOI: https://doi.org/10.25387/g3.16611838.
